# Exploration of intraclonal adaptation mechanisms of *Pseudomonas brassicacearum* facing cadmium toxicity

**DOI:** 10.1111/j.1462-2920.2007.01394.x

**Published:** 2007-11

**Authors:** Delphine Pagès, Lisa Sanchez, Sandrine Conrod, Xavier Gidrol, Agnes Fekete, Philippe Schmitt-Kopplin, Thierry Heulin, Wafa Achouak

**Affiliations:** 1 Laboratoire d'Écologie Microbienne de la Rhizosphère et d'Environnements Extrêmes, CEA, DSV, iBEB, LEMiRE, CNRS, Aix Marseille Universite CEA Cadarache, F-13108 Saint-Paul-lez-Durance, France; 2 Service de Genomique Fonctionnelle, CEA/DSV, Genopole d'Evry 2, rue Gaston Cremieux, CP 22, F-91057 Evry Cedex, France; 3 GSF – National Research Center for Environment and Health, Institute of Ecological Chemistry Ingolstädter Landstraße 1, D-85764 Neuherberg, Germany

## Abstract

*Pseudomonas brassicacearum* forms phenotypic variants *in vitro* as well as *in planta* during root colonization under natural conditions, leading to subpopulations (phase I and II cells) that differ in colony morphology and production of exoenzymes/secondary metabolites. The maximal concentration of cadmium allowing both variants growth was 25 μM; however, phase II cells accumulated fivefold higher Cd than phase I cells, even though both variants showed the same growth rate and kinetics, comprising a long stasis period (50 h). The whole transcriptome analysis of both variants in response to Cd was investigated using the home-made DNA microarrays. This analysis revealed completely different adaptation mechanisms developed by each variant to withstand and grow in the presence of the toxic. A re-organization of the cell wall to limit Cd entrance was noticed for phase I cells, as genes encoding levan exopolymers were downregulated at the expense of an upregulation of genes encoding alginate, and an upregulation of transporters such as *cadA*, and a downregulation of copper transporters. Phase II cells were unable to prevent Cd entrance and recruited genes under the control of *oxyR* and *soxR* regulation to face osmotic and oxidant stresses generated by Cd. Putrescine and spermidine metabolism appeared to play a central role in Cd tolerance. Microarray data were validated by biological analyses such as motility, oxidative stress assay, metabolite profiling with ICR-FT/MS and UPLC, capillary electrophoresis analysis of biogenic amines.

## Introduction

Cadmium is a non-essential but highly toxic metal widespread in the biosphere and known as an important environmental hazard and a potent human carcinogen (Waisberg *et al*., 2003). The exposure of bacteria to cadmium induces expression of several genes involved in metal transport (Nies and Silver, 1995; Hassan *et al*., 1999; Canovas *et al*., 2003), DNA repair, heat shock response and oxidative stress response (Rossbach *et al*., 2000; Banjerdkij *et al*., 2005; Wang and Crowley, 2005). It is of particular environmental interest to understand how soil bacteria react to toxic metals, as they represent a major biomass component in the soil. *Pseudomonas brassicacearum* is a Gammaproteobacterium described as a major root-colonizing population of *Arabidopsis thaliana* and *Brassica napus* (Achouak *et al*., 2000; Persello-Cartieaux *et al*., 2001). The strain NFM421 undergoes phenotypic variation when grown on rich media (Chabeaud *et al*., 2001), which leads to the appearance of phase II cells from the original phase I cells and affects colony morphology and production of exoenzymes as lipase and protease. The operon encoding these enzymes has been characterized and has been shown to be under the control of phase variation at the transcriptional level (Chabeaud *et al*., 2001). Phase variation occurs also during *A. thaliana* and *B. napus* root colonization *in vitro* as well as in soil and is likely to be a colonization strategy that may explain the high colonization power of *P. brassicacearum* (Achouak *et al*., 2004). Phase variation is known as an adaptative process by which bacteria undergo frequent and often reversible phenotypic changes resulting from genetic or epigenetic alterations in specific loci of their genomes (Saunders *et al*., 2003). It occurs at a high frequency of > 10^5^ switches per cell per generation (Henderson *et al*., 1999) and can result in reversible ON or OFF switching of traits or in the variation of surface phenotypes. Varying the expression of some structures can allow bacteria to colonize new ecological niches and to escape host defences (Hallet, 2001). Recent reports show that phenotypic variation is involved in the production of exoenzymes, production of secondary metabolites and affects colonization behaviour and biocontrol activity of rhizosphere bacteria (Chabeaud *et al*., 2001; Chancey *et al*., 2002; Sánchez-Contreras *et al*., 2002; van den Broek *et al*., 2003; Achouak *et al*., 2004). Phase variation can have a broad impact on the ecology of bacteria, in the presence of environmental stresses. The aim of this study was to determine Cd adaptation behaviour of each *P. brassicacearum* NFM421 variant. In this article, we demonstrated that even though they tolerated the same dose and showed the same long stasis period, the original phase I cells accumulated fivefold lower amounts of Cd than the corresponding variant. In order to gain more insight on Cd adaptation mechanisms of each variant, DNA microarrays were used to study global gene expression changes towards metal stress (Hu *et al*., 2005; Wang and Crowley, 2005). DNA microarrays of the non-sequenced genome of this rhizobacterium were manufactured using an original method and used to explore how each phenotypic variant type accommodates and adjusts its physiology in response to cadmium.

## Results and discussion

### Effect on bacterial growth and Cd accumulation

**Fig. 1:**
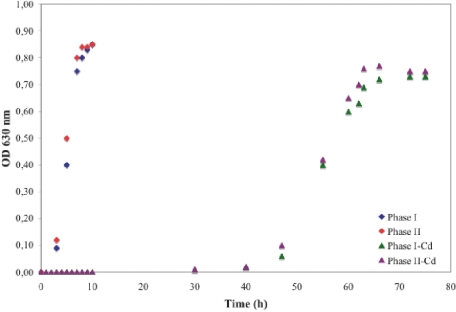
Growth of *P. brassicacearum* phase I and II cells in the presence or absence of the maximal tolerated Cd dose (25 μM).

The maximal concentration of Cd allowing the growth of *P. brassicacearum* NFM421 variants phase I and phase II cells was determined by their growth in the presence of different doses of Cd. No growth was observed at Cd concentrations higher than 25 μM. When grown with 25 μM CdCl_2_, both phase cells of strain NFM421 showed a long stasis period from 5 h in control treatment to 50 h after Cd exposure, and then resumed growth (Fig. 1). The long stasis period may be required for the adjustment of cell physiology to limit the distribution of Cd within the cell or to repair damages that were induced. Such behaviour has been previously reported for *Escherichia coli* (Mitra *et al*., 1975). When Cd-grown bacteria were cultivated directly on Cd-containing medium or after a growth on Cd-free medium, the lag phase was shortened to 26 h, but remained much longer than the lag phase of the control in Cd-free medium (about 5 h). These data disallowed the selection of mutants during the long lag phase of 50 h. Survival test was realized after application of the 25 μM CdCl_2_ and no cell mortality was observed, indicating that Cd had no bactericide effect but bacteriostatic effect. The amount of Cd accumulated by *P. brassicacearum* phase II cells and I, was measured 3 days after Cd exposure by ICP-OES in each variant. Surprisingly, even though both phase I and II cells resumed growth after the same stasis period, we found that phase II cells contained a fivefold higher concentration of Cd (7.5 ± 2.7 mg g^−1^ dry mass) compared with phase I cells (1.5 ± 0.4 mg g^−1^ dry mass). This result prompted us to investigate a comprehensive, general description of the molecular response to Cd mounted by each variant.

### Microarray conception

DNA microarray technologies offer an opportunity to look at changes in the expression of thousands of genes simultaneously under different physiological conditions (DeRisi *et al*., 1997; DeRisi and Iyer, 1999). Because the *P. brassicacearum* genome is not sequenced, a protocol was set up to generate a DNA array specific to this bacterium. As spotted DNA fragment size is a critical parameter for experimental efficiency (both sensitivity and specificity of hybridizations), we therefore used a technique of DNA sonication in order to obtain fragments between 0.5 and 2 kb in length. This technique allowed us to obtain a genomic library containing 12 000 DNA fragments. Among these, 7200 (1 kb average fragment length) were selected for purity and quantity after control on agarose gel and constituted the final *P. brassicacearum* NFM421 genomic library. In order to validate the bank before printing, 150 fragments were randomly chosen among the 7200 and sequenced. After sequence analyses, a classification was realized, based on functional categories using databases (http://www.genome.ad.jp/kegg, http://metacyc.org/), and was in agreement with functional classes of predicted *Pseudomonas aeruginosa* genes (Stover *et al*., 2000). The 7200 fragments that constitute the low redundant (3%) genomic library, as well as 580 controls and identified genes (i.e. *rpoS*, *lysA* …) specifically amplified were then spotted on GAPSII (Corning) glass slides.

### General overview of transcriptomic response of the two *P. brassicacearum* variants to Cd

**Fig. 2:**
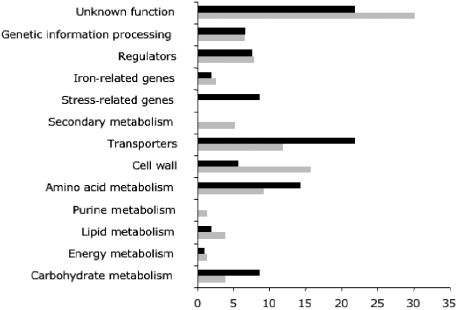
Overview of cadmium response in original phase I cells (black) and the corresponding variant phase II cells (grey). Genes selected by microarray analysis and specifically regulated by each bacterial phase were sequenced and classified according to their putative function. The horizontal axis represents the percentage of genes modulated by Cd.

‘Home-made arrays’ were used to compare the Cd response of *P. brassicacearum* phase II and I cells. After hybridization arrays (see Fig. S1), we sequenced the spots showing significant (*P* ≤ 0.05) modulation of at least twofold up- or downregulation. In total, 146 (54/92, induced/repressed) and 175 (75/100, induced/repressed) genes in phase I and II cells, respectively (see Table 1), exhibited significant differential expression (*P* ≤ 0.05) at a twofold or greater level in at least three of the four hybridizations (see Fig. S1). Among genes that showed a significant differential expression, only 68 were commonly modulated in both phase I and II cells after Cd exposure. We focused analyses on specific genes modulated by each variant. We grouped the Cd-modulated genes for each variant (80 and 76 in phase I and II respectively) according to their putative function (Fig. 2) (http://www.genome.ad.jp/kegg, http://metacyc.org/) and observed that in phase I cells the first barrier to limit Cd distribution into the cell is re-organized, as more genes involved in cell wall composition (16%) are modulated in comparison with the corresponding phase II cells (6%). Interestingly, a global overview of the differences between the two bacterial phases revealed that some stress-related genes were modulated in response to Cd only in phase II cells (Fig. 2).

**Table 1:** Results of microarray analysis.

	Fold change	
	
	I	II
Carbohydrate metabolism		
**43F4 Succinyl CoA synthetase alpha chain sucC (TCA cycle)**	**0.49 ± 0.09**	
**55G5 Succinyl CoA synthetase alpha chain sucD (TCA cycle)**	**0.48 ± 0.04**	
63F9 PEPC (pyruvate metabolism)	0.37 ± 0.09	
**35G10 Glyceraldehyde 3-phosphate dehydrogenase *gapA* (glycolysis)**		**4.11 ± 1.93**
119F9 3-Hexulose 6-phosphate synthase *hxlA* (pentose glucuronate interconversion)		2.82 ± 0.24
46F3 Starvation-sensing protein RspA (galactose metabolism)		2.28 ± 0.36
86G3 Acetate CoA ligase (pyruvate metabolism)		2.30 ± 0.84
82H3 iolC protein (inositol metabolism)		3.87 ± 2.61
20G10 Iron-containing alcohol dehydrogenase (galactose metabolism)		2.40 ± 0.75
**77C5 Periplasmic trehalase precursor *treA* (starch and sucrose metabolism)**		**0.35 ± 0.06**
43B5 Sorbitol dehydrogenase *polS* (fructose mannose synthesis)		0.45 ± 0.08
84D4 Polysaccharide deacetylase (amino sugar metabolism)		0.49 ± 0.07
16G9 Quinoprotein glucose dehydrogenase (pentose phosphate pathway)	2.37 ± 0.40	4.61 ± 0.58
58C8 Acetyl CoA carboxylase *accA accD* (pyruvate metabolism)	0.40 ± 0.13	0.53 ± 0.09
10A10 Acetyl CoA ligase *fadDx* (pyruvate metabolism)	0.32 ± 0.19	0.50 ± 0.06
Energy metabolism (oxidative phosphorylation)		
18H6 NADH quinone oxidoreductase, B subunit *nuoB*	0.40 ± 0.05	
5E6 cbb3-type cytochrome oxidase		0.49 ± 0.10
15F9 NADH quinone oxidoreductase, subunit 5	2.41 ± 0.37	4.56 ± 0.66
15G9 Cytochrome B561	2.62 ± 0.62	5.80 ± 0.70
13D7 Electron transfer flavoprotein, beta subunit	0.52 ± 0.09	0.49 ± 0.03
Lipid metabolism		
24D6 Glycerophosphoryl diester phosphodiesterase	2.40 ± 0.21	
64C8 3-Hydroxyacyl-CoA dehydrogenase *fadB2x*	0.48 ± 0.05	
94D4 Enoyl CoA hydratase/carnithine racemase	0.46 ± 0.13	
**19E10 PAP2 (acid phosphatase) superfamily**		**2.44 ± 0.97**
**12D10 PAP2 (acid phosphatase) superfamily**		**2.19 ± 0.81**
14B6 3-Ketoacyl-CoA thiolase *fadA*	0.44 ± 0.19	0.46 ± 0.07
38F10 Acyl CoA dehydrogenase	0.39 ± 0.21	0.50 ± 0.16
34F7 Short-chain dehydrogenase/reductase SDR	3.10 ± 1.75	4.46 ± 1.70
Purine metabolism		
3A11 GMP reductase	0.26 ± 0.23	
Amino acid metabolism		
10D12 Glucosamine-fructose 6-phosphate aminotranferase	2.21 ± 0.65	
28E2 Glucosamine 6-phosphate synthetase	2.05 ± 0.08	
9C4 Threonyl-tRNA synthetase	2.50 ± 0.47	
71E5 3-Methylcrotonyl-CoA carboxylase, beta subunit	0.28 ± 0.27	
17A8 Arginine deiminase	0.39 ± 0.09	
**74G2 Histidine ammonia lyase *hutH2***	**0.36 ± 0.11**	
**99H9 Urocanase *hutU***	**0.39 ± 0.27**	
**48D11 Acetylornithine deacetylase**		**5.50 ± 4.06**
15B2 Diaminopimelate epimerase *dapF*		2.36 ± 0.28
34B12 Glutamate dehydrogenase/leucine dehydrogenase		2.27 ± 0.73
**57A2 Ornithine carbamoyl transferase**		**0.41 ± 0.05**
**107E4 Succinyl glutamate desuccinylase**		**0.45 ± 0.08**
**25F4 4-Aminobutyrate aminotransferase *gabT***		**0.28 ± 0.05**
**83 E9121 B9 39C9 Succinate semialdehyde dehydrogenase *gabD***		**0.30 ± 0.07**
37C4 76C5 7C10 Glutamine synthetase		0.30 ± 0.08
74B11 Predicted glutamine amidotransferase		0.31 ± 0.10
**46D11 Glutamate-5-kinase, Pro-B related**		**0.47 ± 0.03**
16H8 Homogentisate 1,2-dioxygenase		0.28 ± 0.21
22A8 4-Hydroxyphenylpyruvate dioxygenase		0.46 ± 0.08
77A6 Anthranilate/para-benzoate synthase		0.46 ± 0.12
100B10 Dihydropicolinate synthase		0.31 ± 0.11
54C2 Zn-dependent dipeptidase		0.38 ± 0.04
14C8 Glutamyl- and glutaminyl-tRNA synthetase	0.30 ± 0.07	0.23 ± 0.06
**100C10 24C2 29F3 NAD-glutamate dehydrogenase**	**0.29 ± 0.08**	**0.28 ± 0.07**
84E7 NAD-glutamate dehydrogenase	0.34 ± 0.06	0.24 ± 0.02
76E2 Threonine synthase	0.49 ± 0.08	0.45 ± 0.03
16B9 Glycine dehydrogenase	0.32 ± 0.20	0.39 ± 0.14
21A11 Succinyl arginine dehydrolase *astB*	0.21 ± 0.08	0.14 ± 0.01
24B6 Succinyl arginine dehydrolase	0.37 ± 0.05	0.37 ± 0.07
19H2 NAD-dependent aldehyde dehydrogenase	0.24 ± 0.04	0.26 ± 0.03
20F2 NAD-dependent aldehyde dehydrogenase	0.22 ± 0.11	0.23 ± 0.07
*lysA*	0.47 ± 0.04	0.39 ± 0.24
Cell wall		
**9H1 Alginate biosynthesis algA**	**2.05 ± 0.59**	
105D11 Pilus assembly protein, major pilin A	2.52 ± 0.47	
2H7 Predicted periplasmic/secreted protein	0.16 ± 0.15	
112A3 Peptidase U32 family	0.43 ± 0.20	
**17C9 Bacterial transferase hexapeptide-like protein WbpD**	**0.50 ± 0.04**	
**35H4 LPS biosynthesis protein WbpG**	**0.50 ± 0.14**	
**12A11 Probable oxidoreductase WbpB**	**0.50 ± 0.10**	
104D1 PHA synthase I	0.45 ± 0.20	
21G1 Calcium-binding outer membrane-like protein	0.49 ± 0.07	
15H10 Binding protein-dependent transport system inner membrane	0.48 ± 0.11	
**20F8 CopC protein**	**0.46 ± 0.20**	
42G6 Sucrose 6-phosphate hydrolase (starch and sucrose metabolism)	0.32 ± 0.17	
**75H10 Peptidase A24A, prepilin type IV**		**2.29 ± 0.24**
**22C3 Outer membrane protein**		**2.47 ± 0.85**
**68D9 Predicted outer membrane lipoprotein**		**2.18 ± 0.49**
**27D10 Putative lipoprotein**		**2.29 ± 0.32**
**26C9 Membrane carboxypeptidase/penicillin-binding protein**		**0.32 ± 0.05**
**23H10 Flagellar basal-body rod protein *flgC***		**0.48 ± 0.14**
**106C11 22E4 Alginate biosynthesis alg8**	**2.55 ± 0.30**	**2.60 ± 0.60**
12F10 Levansucrase (starch and sucrose metabolism)	0.30 ± 0.03	0.39 ± 0.06
Transporters		
7C12 Pyrimidine nucleoside transporter	7.76 ± 3.90	
31A1 ABC transporter like	2.25 ± 0.85	
32H6 ABC-type spermidine/putrescine transport system	3.07 ± 1.60	
**7F12 ABC-type spermidine/putrescine-binding periplasmic protein**	**2.50 ± 0.82**	
**35B11 ABC-type sugar transport system, periplasmic component**	**2.33 ± 0.30**	
33H6 TonB	2.20 ± 0.40	
45E8 13E8 Mannitol transporter, periplasmic binding protein	0.33 ± 0.13	
29B3 Putative cyanate transporter	0.26 ± 0.02	
**14E8 Copper-transporting ATPase**	**0.35 ± 0.12**	
33G5 15B11 Predicted permease		2.24 ± 0.20
77D6 Permease MFS		3.20 ± 0.48
72C6 Predicted Na^+^-dependent transporter		2.16 ± 0.56
**50E3 ABC-type Mn^2+^/Zn^2+^ transport system**		**2.21 ± 0.58**
**18D11 ABC-type cobalamin/Fe^3+^ siderophore transport system**		**2.25 ± 0.49**
63D7 43F5 spermidine/putrescine-binding periplasmic protein		2.20 ± 0.70
**27C8 GABA permease**		**0.47 ± 0.09**
32F5 Permease MFS		0.30 ± 0.08
62B12 Dipeptide ABC transporter, periplasmic peptide-binding component		0.47 ± 0.04
23E6 ABC transporter-like, periplasmic component		0.22 ± 0.13
2D4 ABC-type sugar transport system		0.50 ± 0.12
32G11 Amino acid ABC transporter		0.46 ± 0.13
12B5 Amino acid ABC transporter		0.31 ± 0.15
95D3 Amino acid ABC transporter		0.49 ± 0.05
48F9 Amino acid ABC transporter		0.49 ± 0.12
84E6 Amino acid ABC transporter		0.48 ± 0.13
TRAP-type C4-dicarboxylate transport system		0.48 ± 0.11
**47G7 Putrescine-binding protein of putrescine transport system *potF1***		**0.27 ± 0.05**
**79H10 25A11 29C5 30E6 Putrescine-binding protein of putrescine transport system *potF2***		**0.30 ± 0.06**
**14A1 22A7 Transmembrane protein of putrescine transport system *potH***		**0.32 ± 0.07**
**22A6 Transmembrane protein, nucleotide-binding domain *potH potG***		**0.42 ± 0.11**
73G3 General substrate transporter, TonB box		0.46 ± 0.08
79E8 OmpA family protein		0.42 ± 0.23
**16C6 86G11 *cadA***	**20.15 ± 2.06**	**34.35 ± 4.74**
42E6 ABC-type metal ion transport system	2.11 ± 0.55	2.71 ± 0.44
**98F4 Spermidine/putrescine-binding periplasmic protein**	**6.34 ± 3.66**	**8.11 ± 3.43**
**71C8 Sulfate permease MFS family**	**3.11 ± 0.88**	**2.25 ± 0.36**
15E9 Formate/nitrite transporter family	2.13 ± 0.16	3.45 ± 0.63
134D10 ABC transporter, permease protein *braD*	0.37 ± 0.17	0.36 ± 0.12
59H9 ABC transporter, periplasmic peptide-binding protein	0.19 ± 0.11	0.11 ± 0.01
4E5 ABC-type histidine transport system	0.54 ± 0.05	0.49 ± 0.09
75A9 ABC-type nitrate/sulfonate/bicarbonate transport system	0.40 ± 0.12	0.33 ± 0.06
67F10 79E9 13C8 ABC transporter, periplasmic component	0.31 ± 0.10	0.26 ± 0.05
24F5 ABC transporter, periplasmic component	0.29 ± 0.07	0.12 ± 0.05
23H8 39G3 ABC transporter, periplasmic component	0.37 ± 0.03	0.21 ± 0.14
78C4 ABC transporter, probable binding protein component	0.13 ± 0.06	0.16 ± 0.07
45E2 Major facilitator family transporter	0.25 ± 0.10	0.33 ± 0.05
4B10 83G1 96A5 Permease of the MFS	0.32 ± 0.08	0.43 ± 0.09
74B7102A11 Permease of the MFS	0.22 ± 0.15	0.14 ± 0.05
113A2 Probable porin	0.24 ± 0.07	0.24 ± 0.02
**83G4 1H11 ABC-type proline/glycinebetaine transport system**	**2.8 ± 0.90**	**0.52 ± 0.09**
Secondary metabolites biosynthesis		
**115G12 6H7 *phlA***	**6.5 ± 2.00**	
**97D4 *phlB/C***	**3.5 ± 1.40**	
**39E1 85A5 *phlD***	**4.5 ± 1.80**	
31C4 Polyketide synthase	0.40 ± 0.1	
Stress-related genes		
**74C6 Glucose 6-phosphate-1-dehydrogenase**		**6.00 ± 4.11**
**17D4 Universal stress protein UspA**		**3.86 ± 1.70**
**72F10 Heat-shock protein HSP20 family protein**		**3.15 ± 1.10**
113A8 Selenocysteine lyase *sufS*		2.98 ± 1.48
**71B7 Sarcosine oxidase, alpha subunit *soxA***		**3.49 ± 2.18**
**90D2 Heat-shock protein HslV**		**0.35 ± 0.14**
**90C6 Heat-shock protein HslV**		**0.25 ± 0.02**
**9A1 Heat-shock protein HtpG**		**0.41 ± 0.20**
**88F8 Alkyl hydroperoxide reductase, subunit C *ahpC***		**0.32 ± 0.12**
**29B7 inaA protein**	**2.29 ± 0.65**	**2.44 ± 0.79**
**100C9 Cold-shock protein CapB**	**0.46 ± 0.07**	**0.47 ± 0.10**
Iron-related genes		
13A11 Pyoverdine synthetase A	0.49 ± 0.12	
19H6 4Fe-4S ferredoxin, iron-sulfur binding	0.37 ± 0.08	
10D2 Polyferredoxin		2.55 ± 0.21
51H9 Isochorismatase family protein		0.43 ± 0.08
Regulators		
126C1 DNA-binding response regulator, LuxR family	2.34 ± 0.82	
67B8 Putative sensor protein RstB	2.81 ± 0.74	
5F8 Histidine kinase colS	2.00 ± 0.50	
133G4 Transcriptional regulator PcaQ	0.47 ± 0.12	
10C11 Transcriptional regulator LysR family	0.34 ± 0.11	
13A10 Putative transcriptional regulator	0.44 ± 0.14	
120C8 RpoC		3.57 ± 1.20
45C12 Response regulator containing CheY-like receiver		3.15 ± 1.14
34F8 Sensor histidine kinase/response regulator		3.02 ± 0.95
**108A5 Transcriptional regulator Crp/Fnr family**		**2.12 ± 0.59**
114A4 Transcriptional regulator MerR family		2.04 ± 0.54
94G12 Transcriptional regulator AraC family		0.48 ± 0.10
17H6 Sensory box histidine kinase PAS/PAC domain		0.41 ± 0.12
24D7 GGDEF domain		0.49 ± 0.14
75C3 Two-component response regulator	2.46 ± 0.51	4.02 ± 1.18
112C7 Sigma-54-dependent transcriptional regulator	0.30 ± 0.09	0.45 ± 0.10
Genetic information processing		
5C1 Ribosomal protein S1	2.11 ± 0.35	
2OD2 HupN histone-like protein	2.04 ± 0.34	
7H12 DNA dependent DNA polymerase, beta chain	3.36 ± 2.02	
21C2 30S ribosomal protein S11	0.50 ± 0.10	
23D12 Putative DNA methyltransferase	0.40 ± 0.03	
73H9 Cell division protein FtsL		3.02 ± 1.04
16E6 Translation initiation factor IF-1		2.46 ± 0.86
82D9 Putative transposase TnpG		2.57 ± 0.54
119F7 Ribosome modulation factor		2.17 ± 0.50
45D1 Ribonuclease HI		0.5 ± 0.04
81A11 S1 RNA-binding domain protein		0.48 ± 0.10
17F1 GyrB type II topoisomerase, beta subunit		0.4 ± 0.12
45H1 Translation elongation factor G	2.56 ± 0.57	2.45 ± 0.73
23F1 Topoisomerase IA	0.46 ± 0.08	0.43 ± 0.12
***rpoS***	**0.41 ± 0.13**	**4.18 ± 1.55**
110F1 Putative DNA repair protein RadA	2.87 ± 0.91	0.36 ± 0.26
Unknown function		
54B8 Rhs element Vgr protein	0.39 ± 0.06	
68E11 Predicted aminopeptidase	0.28 ± 0.01	
15B4 Aerobic-type carbon monoxide dehydrogenase, CoxL/CutL homologue		2.99 ± 0.41
108E7 Probable protease		2.17 ± 0.78
34A9 Protozoan/cyanobacterial globin family protein		2.16 ± 0.59
33C2 Predicted membrane protein		2.78 ± 1.00
131E4 15G11 GTP-binding protein EngB		2.7 ± 0.75
133F11 Amidase		0.36 ± 0.06
108D10 fmtB-like protein		0.38 ± 0.13
28F10 Mu-like prophage tail sheath protein gpL		0.38 ± 0.05
32F7 Predicted membrane protein	21.93 ± 9.82	92.66 ± 28.65
33C6 Phage-related protein	12.91 ± 3.80	33.35 ± 14.50
86C3 Penicillin amidase V	2.71 ± 0.80	3.09 ± 0.79
72G4 Putative membrane protein	0.35 ± 0.16	0.32 ± 0.11
77 E7 Putative ATP/GTP-binding protein	0.49 ± 0.13	0.40 ± 0.09
100F9 Uncharacterized protein homologue of lactam utilization	0.11 ± 0.01	0.18 ± 0.05
9D3 PspB homologue (serine protease)	0.31 ± 0.10	0.35 ± 0.05
No homology		
84G2 No homology	3.47 ± 0.40	
5D12 No homology	2.10 ± 0.41	
79B10 No homology	0.49 ± 0.07	
73E8 No homology	0.46 ± 0.14	
71B6 No homology	0.49 ± 0.14	
27D12 No homology	0.40 ± 0.17	
108F6 No homology		2.84 ± 1.53
21D3 No homology		0.42 ± 0.12
68H2 No homology		0.27 ± 0.03
29E6 No homology		0.24 ± 0.04
23F2 No homology	0.49 ± 0.13	0.43 ± 0.19
81B3 No homology	0.41 ± 0.11	0.44 ± 0.15
26E9 No homology	0.45 ± 0.11	0.50 ± 0.05
30C4 No homology	0.48 ± 0.14	0.47 ± 0.09
Hypothetical proteins		
73F9 Unknown protein	4.31 ± 0.96	
77F4 Unknown protein	3.61 ± 1.25	
38E6 Unknown protein	2.77 ± 0.34	
76E8 Conserved hypothetical protein	2.46 ± 0.59	
38E1 Hypothetical protein	2.27 ± 0.20	
17D11 Unknown protein	0.47 ± 0.09	
19F8 Unknown protein	0.43 ± 0.17	
49A7 Unknown protein	0.48 ± 0.09	
84A10 Conserved hypothetical protein	0.44 ± 0.14	
16D8 Hypothetical protein	0.32 ± 0.12	
16B2 Hypothetical protein	0.48 ± 0.13	
55D1 Hypothetical protein	0.45 ± 0.08	
75E6 Hypothetical protein	0.41 ± 0.08	
76F7 Hypothetical protein	0.48 ± 0.12	
90A3 Hypothetical protein	0.43 ± 0.22	
2C9 Unknown protein		3.44 ± 1.17
34H9 Conserved hypothetical protein		3.12 ± 1.15
36B10 Unknown protein		3.07 ± 0.82
53G10 Hypothetical protein		2.93 ± 1.38
55E9 Hypothetical protein		2.51 ± 0.78
133E2 Unknown protein		2.38 ± 0.87
28D9 Hypothetical protein		2.25 ± 0.45
59D5 Hypothetical protein		2.18 ± 0.56
15H9 Hypothetical protein		2.08 ± 0.60
97D3 Unknown protein		0.32 ± 0.07
56D2 Hypothetical protein		0.47 ± 0.14
11F5 Hypothetical protein	2.31 ± 0.71	2.54 ± 1.02
45A12 Hypothetical protein	3.75 ± 2.30	3.20 ± 1.09
74G8 Hypothetical protein	2.00 ± 0.38	2.74 ± 1.19
20F7 Hypothetical protein	10.85 ± 3.75	13.34 ± 4.96
17C6 Hypothetical protein	10.11 ± 3.33	11.41 ± 2.80
73H11 Unknown protein	2.30 ± 0.73	3.55 ± 1.08
131C9 Unknown protein	8.27 ± 2.81	14.68 ± 5.20
13C10 Hypothetical protein	0.40 ± 0.08	0.49 ± 0.13
89F1 Conserved hypothetical protein	0.44 ± 0.13	0.30 ± 0.07
20F4 Conserved hypothetical protein	0.29 ± 0.14	0.33 ± 0.10
18A2 Conserved hypothetical protein	0.36 ± 0.04	0.24 ± 0.03
106A9 Hypothetical protein	2.27 ± 0.50	0.48 ± 0.10

Fold change (*P* < 0.05) is indicated for original phase (I) and the corresponding variant phase (II) cells. This factor is a mean of the different hybridization experiments. The codes in the first column are the clone numbers. Lines in bold indicate genes discussed in this article.

### Specific response in phase I cells

#### 

##### Re-organization of the cell wall

The whole expression profile of phase I cells revealed that genes involved in cell wall structures were modulated, likely to prevent Cd entrance. Actually, bacterial exopolymers directly interact with the environment. Phase I cells downregulated the expression of the *sacA* and *sacB* genes (12F10, Table 1) responsible of levan (fructose polymer) synthesis, as well as genes of a PHA (polyhydroxyalkanoate) synthase, and may replace them by alginate, as indicated by upregulation of the expression of the *algA* (106C11 and 22E4) genes (Table 1). Competition between PHA accumulation and alginate biosynthesis was previously shown to occur in *P. aeruginosa* (Pham *et al*., 2004).

Transcriptomic analysis revealed a downregulation of the expression of three genes involved in LPS biosynthesis: *wbpB* (12A11), *wbpD* (17C9) and *wbpG* (35H4) (Table 1). Merkx-Jacques and colleagues (2004) suggested that WbpB might synthesize sugar derivatives dedicated to the glycosylation of proteins involved in lipopolysaccharide (LPS) and flagellum synthesis. Recently, it was shown that WbpD is an *N*-acetyltransferase playing an important role for B-band O-antigen biosynthesis in *P. aeruginosa* PA01 (Wenzel *et al*., 2005). As LPS is a major lipid compound found in the outer membrane of Gram-negative bacteria and serves as a barrier to many antibiotics (Rosenfeld and Shai, 2006), it represents the primary site of interaction between many toxic metals and the cell. Indeed, changes in lipid composition may enable microorganisms to maintain membrane functions while facing environmental fluctuations.

##### Optimization of transport system

An important proportion of genes (13%), whose expression was modulated in response to CdCl_2_ exposure, encode ABC transporters (Fig. 2). Induction was noticed for the periplasmic components of ATP-binding proteins of ABC-type transporters involved in the transport of sugar (35B11), spermine/putrescine (7F12) and proline/glycine-betaine (83G4 and 1H11) (Table 1). In general, Gram-negative bacteria achieve high intracellular concentrations of proline and glycine-betaine during osmotic or metal stress through increased transport (Csonka, 1989; Velasco-Garcia *et al*., 1999; Caldas *et al*., 1999; Sharma and Dietz, 2006). Our results, underlying common pathways between heavy metal and osmotic stresses, are in accordance with a recent study on *E. coli*'s response to Cd toxicity (Wang and Crowley, 2005). Among the specific response developed by phase I cells, the decreased expression of *hutH2* (74G2) and *hutU* (99H9) may lead to reduced histidine degradation (Table 1), as described in *Bacillus subtilis* after Cd exposure (Moore *et al*., 2005). Histidine has been shown to play a role in metal binding (Sharma and Dietz, 2006).

The mRNA level of a copper-transporting ATPase, known to be involved in Cu uptake (Rensing *et al*., 2000), decreased after Cd exposure in phase I cells (14E8, Table 1). A repression of this transporter may prevent Cd entry into bacterial cells. This result is in agreement with the downregulation of the copper resistance protein encoded by the *copC* gene (20F8, Table 1). Moreover, a high induction of the gene encoding the P-type ATPase CadA involved in Cd efflux corroborates the low Cd content of phase I cells.

##### Cd induces a switch to anaerobic metabolism

In response to Cd exposure, phase I cells specifically downregulated expression of two genes involved in the TCA cycle: succinyl-CoA synthetase alpha chain *sucC* (43F4) and beta chain *sucD* (55G5) (Table 1). The repression of the *sucABCD* operon was previously reported in *E. coli* in response to Cd toxicity (Wang and Crowley, 2005) and was interpreted as a switch from the citric acid cycle to its branched or non-cyclic anaerobic metabolism. Indeed, cellular metabolism might be altered to promote energy conservation and prevent free radical components production.

##### Secondary metabolites

**Fig. 3:**
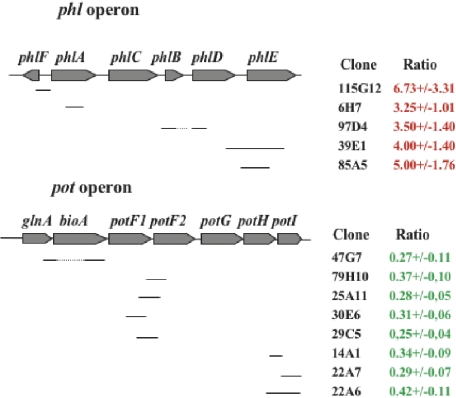
Identification of two operons involved in PHL synthesis (*phl* operon) and putrescine uptake (*pot* operon), respectively, upregulated (red) in phase I cells and downregulated (green) in phase II cells in response to heavy metal toxicity. The ratios of gene expression obtained in the microarray analysis are indicated for each clone. A thin bar represents each cloned DNA fragment.

**Fig. 4:**
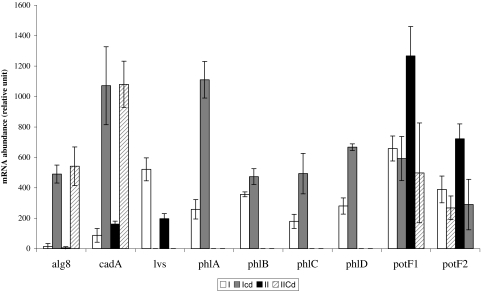
Expression analysis by semi-quantitative RT-PCR of *cadA*, *lvs*, *alg8*, *phlA*, *B*, *C*, *D*, *potF1* and *potF2* genes. Amplification signals of cDNA from phase I or II cells, cultivated with or without added CdCl_2_, were quantified and normalized using the control gene.

**Fig. 5:**
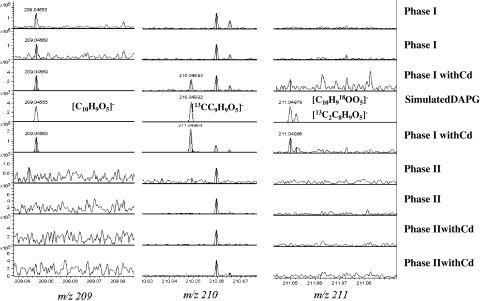
ICR-FT/MS of the four extracts (phase I and II with and without CdCl_2_) in replicates with the simulated spectrum of DAPG showing the various isotopes used for elemental composition confirmation.

Surprisingly, the mRNA level of *rpoS* decreased in phase I cells in response to Cd (Table 1), whereas it is considered as a stress sigma factor (Heeb *et al*., 2005). However, the presence of this metal led to an overexpression of the genes phlA (115G12 and 6H7), phlB/C (97D4) and phlD (39E1 and 85A5), involved in 2,4-diacetylphloroglucinol (DAPG) biosynthesis and known to be organized in an operon (Bangera and Thomashow, 1996; 1999; Delany *et al*., 2000) (Table 1, Fig. 3). Duffy and Defago (1999) showed that Zn^2+^ stimulated DAPG overproduction. In order to gain more insight, it would be interesting to pursue a mutagenesis approach to determine whether a mutant affected in DAPG production is impaired in Cd tolerance or whether DAPG contributes to chelating Cd and its efflux from the cells. It is worth noting that in *P. brassicacearum*, the expression of the *phl* genes is under phase-variable control, as shown using reverse transcription polymerase chain reaction (RT-PCR) experiments (Fig. 4). Moreover, profiling the metabolites with ultrahigh resolution mass spectrometry showed that phase II cells did not show any trace of DAPG. DAPG was present in phase I cells extracts and its concentration increased 100-fold in the same phase in presence of Cadmium. Figure 5 shows the spectra of all modalities (replicates) with the simulated spectrum of DAPG showing the isotopic traces (one ^13^C, two ^13^C and ^18^O) used for elementary composition confirmation of that component (C_10_H_10_O_5_).

### Phase II cells response to CdCl_2_

#### 

##### Transporters

Phase II cells specifically modulated the expression of 23 membrane transporters (22% of regulated phase II genes, Fig. 2 and Table 1). Among the ABC transporters upregulated in response to Cd, an Mn^2+^/Zn^2+^ transporter (50E3, Table 1) and a cobalamin/Fe^3+^/siderophore transporter (18D11, Table 1) were discerned. It is generally assumed that Cd enters bacterial cells through the transport systems for essential divalent cations such as Mn^2+^ or Zn^2+^ (Hao *et al*., 1999). These data are in agreement with the fivefold higher Cd content of phase II cells compared with phase I cells. Microarray data suggested that phase II cells might deal with Cd toxicity through the regulation of genes required to face osmotic and oxidant stresses.

##### Cadmium generates an oxidant stress

The whole profile analyses indeed revealed fundamental changes in the expression level of stress related genes in phase II cells: analyses pursued in these phase II cells showed modulation of the expression of several hydrogen peroxide stress-related genes in response to Cd. The gene *rpoS*, whose expression is repressed in phase I cells, is fourfold induced in the corresponding variant phase II and such overexpression may contribute to counteract the oxidative stress (Heeb *et al*., 2005) generated by cadmium. The gene *inaA* (29B7, Table 1), 2.4-fold overexpressed, is a SoxR/S regulated gene whose expression is triggered by hydrogen peroxide (Rosner and Slonczewski, 1994; Manchado *et al*., 2000). Phase II cells showed a fourfold enhanced expression of the *gapA* gene (35G10, Table 1). This gene encodes a key enzyme of the glycolytic and gluconeogenesis pathways and was shown to be significantly increased in response to H_2_O_2_ (Manchado *et al*., 2000). Moreover, the downregulation observed in our study for the *ahpC* gene (88F8, Table 1) indicates an oxidative stress caused by Cd in phase II cells. Indeed, *ahpC* transcript and protein expression levels are greatly decreased under oxidative stress (Chuang *et al*., 2005). Among other oxidant stress indicators, a significant (more than sixfold) increase in mRNA levels corresponding to a glucose 6-phosphate-1-dehydrogenase (G6PD) was observed (74C6, Table 1). G6PD is mainly associated with the production of NADPH necessary for peroxidase and glutathione detoxification of reactive oxygen species (ROS) (Izawa *et al*., 1998).

Among other stress-related genes whose expression was altered by Cd in phase II cells, we identified genes encoding, heat-shock proteins HSP20 (72F10), two HslV (90D2–90C6), HtpG (9A1), cold-shock protein CapB (100C9) and the universal stress protein UspA (17D4) (Table 1). UspA was recently described as a protective against superoxide stress (Nachin *et al*., 2005). Moreover, an upregulation of *sufS* (113A8) was observed. *sufS* encodes for a cytosolic cysteine desulfurase which mobilizes the sulfur atom from cysteine and provides it to the [Fe-S] cluster. Recently it was discovered that the sufABCDSE operon is induced during exposure to hydrogen peroxide (H_2_O_2_) in *E. coli* (Zheng *et al*., 2001). [Fe-S] clusters may represent Cd targets and *sufS* may repair and maintain clusters integrity. A positive correlation between the Cd and oxidative stress responses was underlined by microarray data. We tested whether Cd treatment sensitizes cells to H_2_O_2_ and to methyl-viologen (paraquat). No significant difference in paraquat sensitivity was observed in phase I and II cells with or without added CdCl_2_. On the other hand, the susceptibility to H_2_O_2_ increased more significantly for phase II cells after exposure to Cd (Table 2). These data suggest that the higher intracellular content of Cd in phase II cells probably mediated an increase in H_2_O_2_-generating compounds.

**Table 2:** Oxidative stress tolerance of *P. brassicacearum* NFM421: sensitivities to H_2_O_2_ and to methyl viologen were evaluated on agar plates of bacteria pre-cultivated in the presence or absence of CdCl_2_ (25 μM) by measuring the size of inhibition halo (in cm).

	Oxidative sensitivity			
	
	H_2_O_2_ tolerance		Paraquat tolerance	
		
Cd concentration in culture medium	I	II	I	II
0	3.2 ± 0.44	3.6 ± 0.35	3.2 ± 0.01	3.1 ± 0.12
25 μM	4.5 ± 0.22	5.2 ± 0.29	3.7 ± 0.32	3.8 ± 0.17

Values are an average of three independent experiments.

##### Polyamines to counter Cd-induced damages

Phase II cells modulated the expression of several genes involved in polyamine metabolism. Microarray analysis allowed the identification of five genes known to be organized in an operon (Fig. 3) and previously studied in other *Pseudomonas* species: an aminotransferase *bioA* (47G7), two putrescine-binding proteins *potF1* (47G7) and *potF2* (79H10, 25A11, 29C5 and 30E6), a nucleotide-binding domain *potG* (22A6) and a transmembrane protein *potH* (14A1, 22A7 and 22A6) (Table 1). Theses genes are involved in putrescine (but not spermidine) uptake. The expression of these genes was repressed in phase II cells in response to Cd, as were two genes implied in putrescine degradation: the 4-aminobutyrate-transaminase *gabT* (25F4) and the succinate-semialdehyde dehydrogenase *gabD* (83E9, 121B9 and 39C9) (Table 1). Interestingly, we observed a fivefold upregulation of the acetyl-ornithine deacetylase *argE* gene (48D11) leading to the production of ornithine, and a downregulation of the ornithine-carbamoyl transferase *argF* gene (57A2) preventing the transformation of ornithine into citruline. It is interesting to notice that ornithine is the direct precursor of putrescine. Thus, these data suggested that the ornithine pool is increased in phase II cells and the putrescine pool generated during the adaptation period to Cd toxicity is preserved. The protective role of polyamines against hydrogen peroxide (H_2_O_2_)-induced oxidative stress has been reported (Jung *et al*., 2003). To validate microarray data concerning polyamines, measurement of biogenic amines was realized by capillary electrophoresis with indirect UV-Vis detection. Results indicated that phase II cells were able to maintain the intracellular pool of putrescine (9.2 and 9.5 mg g^−1^ dry cell weight, control and Cd-treated respectively) and increased significantly the intracellular pool of spermidine (11.5 and 16.3 mg g^−1^ dry cell weight, control and Cd-treated respectively); whereas phase I cells showed a significant decrease of putrescine (15.2 and 6.8 mg g^−1^ dry cell weight, control and Cd-treated respectively) and spermidine (7.7 and 3.8 mg g^−1^ dry cell weight, control and Cd-treated respectively) in response to Cd.

##### Osmoprotection

A downregulation of the trehalase precursor *treA* (77C5) may prevent the degradation of the osmoprotectant trehalose, and allow bacteria to face the osmotic stress. These results are in agreement with the data published by Barra and colleagues (2003) showing that G6PD was required for sucrose and trehalose to be efficient osmoprotectants in *Sinorhizobium meliloti*, and that its role in osmotolerance could be linked to the defence against oxidative stress promoted by osmotic injury. Cadmium induced a modulation of the metabolism of certain amino acids such as glycine, glutamate and tyrosine. In phase II cells the expression of the GABA permease (27C8) was repressed, while the conversion of sarcosine into glycine may be enhanced by upregulation of the *soxA* gene (71B7) and the expression of a glycine dehydrogenase (16B9) involved in glycine catabolism was repressed (Table 1). Several genes encoding enzymes involved in glutamate conversion appear to be downregulated in phase II cells: succinyl-glutamate desuccinylase (107E4), glutamate-5-kinase (46D11) and NAD-glutamate dehydrogenase (100C10, 24C2 and 29F3) (Table 1). These results may indicate an accumulation of glutamate that would serve as osmoprotectant in the variant cells in response to Cd toxicity. Indeed, accumulation of glutamate was previously observed in the bacterial response to osmotic stress (Botsford *et al*., 1994; Aspedon *et al*., 2006). The UPLC analyses showed that phase II cells may maintain their glutamate pool in response to Cd (19 and 26 ± 14% mg g^−1^ without and with Cd respectively). In contrast, in the original phase I cells, a drastic decrease of this pool was observed in the same conditions (88 ± 1% to 30 ± 4% mg g^−1^ without and with Cd respectively).

##### Motility

A downregulation in phase II cells was observed for the *flgC* gene implied in motility. We performed a motility assay of phase II cells in response to Cd, and showed that in the presence of Cd, the bacterial motility was highly diminished witch may contribute to energy economy (Fig. 4).

##### Probable precipitation of Cd

Two genes encoding PAP2 phosphatases were upregulated (2.4- and 2.2-fold increase) after Cd exposure (19E10 and 12D10, Table 1). Type 2 phosphatidic acid phosphatase (PAP2) is an integral membrane protein (Waggoner *et al*., 1999) that catalyses the hydrolysis of lipids. The release of phosphate via the hydrolysis of an organic phosphate has been shown to be an effective method for the precipitation of metals on cell membranes. Macaskie and Dean (1982) isolated a cadmium-resistant strain of *Citrobacter* that precipitated numerous metals, such as cadmium and uranyl ion, as metal phosphates through the use of a membrane-bound acid phosphatase (Macaskie *et al*., 1987; 1992; Macaskie, 1990).

## Semi-quantitative RT-PCR

**Fig. 6:**
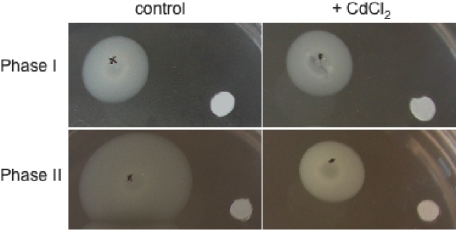
Motility assay on TSB/10 solidified with agar at 0.5 g l^−1^. Phase I and II cells (10 μl) cultivated with or without Cd were spotted on plates and 5 μl of CdCl_2_ 1 M was deposited on a disk.

Microarray data were validated by semi-quantitative RT-PCR using specific primers (see Table S1). The expression level of *cadA*, *lvs*, *alg8*, *phlA*, *B*, *C*, *D*, *potF1* and *potF2* mRNA transcripts in phase I and II cells grown with or without Cd were determined. Results observed by RT-PCR using cDNA from three different RNA batches (than those used in array hybridization) were in accordance with those obtained by array analyses. Results of these experiments, shown in Fig. 6, are means of three independent biological experiments.

## Conclusions

The conception of ‘home-made’ DNA microarrays from a non-sequenced genome constitutes a powerful genomic tool that allowed us to identify for the first time 251 transcripts modulated by Cd in a phase-variable rhizobacterium. The biological significance of our transcriptomic data was strengthened: different genes from the same cluster or metabolic pathway were identified and their modulation factors were closely related, such as the *phl* and *pot* clusters (Fig. 3). Microarray data were also validated by RT-PCR (*sacB*, *algB*, *cadA*, *phl* and *pot* clusters) using specific primers (Table S1) and by biological experiments. This work revealed the originality and the interest to investigate differential adaptative mechanisms to metal toxicity by variants from the same strain. The generation of intraclonal diversity by *P. brassicacearum* allowed us to explore two different modes of Cd tolerance in bacteria. Even though in phase I and II cells showed the same adaptative period of 50 h to adjust their physiology, two different adaptation mechanisms to counteract Cd toxicity were observed. In phase I cells, we could discern the selection of transporters, by a downregulation of copper (and Cd) transporters and by a strong upregulation of efflux system such as *cadA*, and an upregulation of alginate encoding genes at the expense of levan. The upregulation of genes involved in DAPG production was strongly correlated with RT-PCR results and metabolite profiling (ultra-high resolution mass spectrometry). Actually only phase I cells were able to produce DAPG and to increase up to hundreds-fold its synthesis after Cd exposure. The role of DAPG in Cd tolerance merits a deeper investigation. Phase II cells unable to prevent Cd entry, witch generated an oxidative stress, maintained the intracellular pool of putrescine and increased that of spermidine, as underlined by microarrays data and capillary electrophoresis with combination of indirect UV detection. The contradictory expression level of the stress sigma factor *rpoS* in phase I and phase II cells is in agreement with the transcriptome analysis that indicates a much higher stress generated by Cd in phase II cells that contained a higher amount of Cd. This work emphasizes the possibility to explore the whole trancriptome of any environmental bacterial isolate, even if its genome sequence is not yet available. This study gives more insights on the role of phenotypic switching on the ecology of soil borne bacteria in response to environmental stresses.

## Experimental procedures

### Bacterial strains and growth conditions

To determine the effect of Cd exposure on bacterial growth kinetics, *P. brassicacearum* NFM421 (phase I or II cells) were cultivated in 10-fold diluted TSB (TSB/10) (Difco) supplemented or not with different doses of CdCl_2_ at 30°C under shaking. CdCl_2_ (25 μM) corresponded to the maximal dose allowing the growth of both phase I and II cells of *P. brassicacearum* NFM421.

### Production of a *P. brassicacearum* NFM421 DNA microarray

Genomic DNA of phase I cells was extracted with the Nucleospin Macherey-Nagel kit and fragmented by sonication [with runs of 1 min 30 s (7 s ON and 1 s OFF), at 20% amplitude, Bioblock Scientific] to obtain fragments from 0.5 to 2 kb. All fragments were digested for 30 min at 37°C with S1 nuclease to leave in blunt ends. In order to facilitate insertion into pGEM-T Easy Vector (Promega), DNA fragments were incubated in the presence of Goldstar polymerase (Eurogentec) to add dATP nucleotides to the fragment extremity. After transformation, a total of 12 000 clones were harvested and each inserted fragment was amplified using T7 and SP6 primers (Eurogentec). All the 12 000 clones were purified using the Millipore microplates PCR purification system and deposited on 1.5% agarose gels for fragment quantification and purity control (one single fragment). After PCR products analysis, 7200 PCR products were retained for the *P. brassicacearum* NFM421 genomic library. Certain genes that were previously identified in *P. brassicacearum* NFM421 were specifically amplified. Moreover, one 384-well microplate containing PCR-amplified eukaryotic open reading frames (ORFs) was used as a negative control. The DNA spotting was carried out on silane glass slides, at the CEA/SGF laboratory at Evry (France).

### Cell growth and RNA isolation

Phase I and II cells of *P. brassicacearum* NFM421 were cultivated separately in TSB/10 (Difco) supplemented or not with CdCl_2_ (25 μM) at 30°C under shaking until the contents of the flasks reached an optical density at 600 nm of 0.7. Cells were harvested at this time, blocked in bacterial RNA protect (Qiagen) and stored at −80°C until further processing. Total RNA from bacteria was isolated with the Trizol reagent (Invitrogen) and DNAse treatment using the Ambion kit (Ambion, Austin, TX), according to the manufacturer's manual. RNA in samples was quantified spectrophotometrically by measuring extinction at 260 nm and purity was checked by gel electrophoresis.

### DNA microarrays, hybridization and washing

For microarray experiments, synthesis of cDNA from RNA was performed with the CyScribe First-Strand cDNA Labelling Kit (Amersham Bioscience, Little Chalfont, England). In the following description all ingredients are contained in the CyScribe kit unless otherwise noted. Reverse transcription was performed using 20 μg of total RNA (maximally in 10 μl) and 1 μl of random nonamer primers. The volume of the assay mixture was adjusted to 11 μl with RNase-free water, then the assay mixture was incubated for 5 min at 70°C, followed by incubation for 10 min at room temperature to allow the primers to anneal with the RNA. After cooling down to room temperature the reagents for the labelling reaction were added. After the addition of 4 μl of 5× CyScript buffer, 2 μl of 0.1 M DTT, 1 μl of dCTP nucleotide mix, 1 μl of either 0.5 mM Cy3-labelled or Cy5-labelled DCTP (Amersham Bioscience) and 1 μl of CyScript reverse transcriptase (100 U μl^−1^), the final reaction volume was 20 μl. The different labelling is detailed in the supplemental experiment design (Fig. S1). The labelling reaction was performed at 42°C for 1.5 h, followed by RNA degradation and cDNA purification. The RNA was degraded by addition of 2 μl of 2.5 M NaOH, the mixture was then heated at 65°C for 10 min and subsequently neutralized with 10 μl of 2 M Hepes buffer. Purification of labelled cDNA was performed on the column of the CyScribe GFX purification kit (Amersham Biosciences) and the purified cDNA obtained was dried under speed-vacuum and stored at −20°C.

Biological experiments were carried out twice, and one dye swap was realized for each biological experiment (see Fig. S1). Spotted silane glass slides were hydrated under boiling water, and rapidly dried on an 80°C hotplate. Hydrated glass slides were placed in a DNA cross-linker at 260 mJ (Stratalinker) and subsequently washed for 20 min under orbital shaking in a bath containing 150 ml of n-methyl-pyrrolidinone (Sigma), 3 g of succinic anhydride (Sigma) and 17 ml of 0.2 M boric acid (Sigma, pH 8 adjusted with NaOH). Slides were quickly and successively transferred in a water bath, and in a 150 ml of 100% ethanol bath (Prolabo), and immediately dried by centrifugation for 7 min at 500 r.p.m. Treated glass slides were then placed for 30 min at 50°C in pre-hybridization chamber containing a filtered solution of 0.3 g bovine serum albumin (Sigma), 5.25 ml of 20× SSC (Gibco Brl), 300 μl of 10% SDS (Eurobio) and 24.45 ml of ultra-filtrated water. Slides were successively washed for 1 min in 150 ml of ultra-filtrated water and 1 min in 150 ml of isopropanol (Prolabo). A 7 min centrifugation step at 500 pm was performed to dry glass slides, which were then ready for the hybridization experiments. cDNA samples (for detail, see Fig. S1) were re-suspended in 15 μl of Dig-easy (Roche) containing 10 μg of salmon sperm DNA (Sigma), and solutions were warmed for 5 min in boiled water. Ten microlitres of cDNA solution was deposited on glass slides. Hybridizations were performed for 16 h at 42°C in hybridization chambers (Corning). Hybridized glass slides were first washed for 15 min, under orbital shaking and darkness, in 0.1× SSC, 0.1% SDS (700 ml). Then, the slides were washed twice for 10 min in 0.1× SSC (500 ml each), and then dried by centrifugation for 7 min at 500 r.p.m. Hybridized slides were conserved protected from dust and light at room temperature.

### Image and data analysis

Microarray slides were scanned using the Genepix 4000 Scanner (Axon Instruments). Spot intensities and corresponding background signals were quantified with the Genepix software. Further data analysis was performed with the program GeneSpring from Silicon Genetics (Redwood City, CA, USA). The ‘Per spot per Chip: intensity-dependent (lowess) normalization’ and ‘data transformation dye-swap Interpretation’ were realized using the log ratio mode. Induction factors were calculated from the Cy3 and Cy5 signal intensities of the spot. Statistical analyses were then performed on the spreadsheet selected from GeneSpring and genes that were differentially regulated ≥ 2 and ≤ 0.5 (*t*-test, *P* ≤ 0.05) in three on four slides were defined as being statistically different and sequenced. Identification and annotation of genes were realized using blast in NCBI (http://www.ncbi.nlm.nih.gov/BLAST/). Sequences were deposited in GenBank (Accession No. ER896029–ER896296).

### Semi-quantitative RT-PCR

Array data were validated by RT-PCR using specific primers, temperature annealing and cycles number described in Table S1. Total RNA extraction and DNAse treatment were realized as described before. cDNA was prepared from 1 μg of total RNA using the Omniscript RT kit (Qiagen) according to the manufacturer's instructions and normalized using the housekeeping gene 16S rRNA. Different cycles numbers were used in order to determine amplification curve and used the appropriated cycle number, before saturation signal. Amplification products were analysed by 2% agarose gel electrophoresis stained with ethidium bromide and quantified in a Storm 860 phosphorimager with ImageQuant software (Molecular Dynamics, Amersham Bioscience, Orsay, France).

### Analysis of cadmium accumulation

To determine the Cd content of bacterial cells grown in the presence of Cd, an analysis using an inductively coupled plasma atomic emission spectrometry (ICP-OES device; Varian) was performed. The procedure consisted of freeze-drying washed cell pellets (phase I and II) that were grown separately for 3 days without (control treatment) or with CdCl_2_ (25 μM), samples were totally digested by HNO_3_ 70% using microwave and then analysed by ICP-AOS. The Cd concentrations are means (±standard deviations) of results of three independent experiments in each case.

### Oxidative stress assay

Oxidative sensibilities to H_2_O_2_ and to methyl-viologen (paraquat) were performed on TSB/10 agar plates by spreading, bacteria that have been exposed or not to CdCl_2_, and 5 μl of H_2_O_2_ 10 M, or paraquat 200 mM were deposited on a disk in the centre of the plates. Plates were incubated overnight at 30°C. Tolerance values are means of the size of the inhibition halo (in cm) for three independent experiments.

### Motility assays

Motility was determined on plates containing TSB/10 solidified with agar at 0.5 g l^−1^. Phase I and II cells (10 μl) were spotted on plates in the presence of a 5 mm filter disk containing 5 μl of sterilized ultrapure water or of CdCl_2_ 1 M. Plates were incubated overnight at 30°C.

### Analysis of biogenic amines

For the quantification of biogenic amines from the Cd-treated cells, capillary electrophoresis (CE) with combination of indirect UV detection was used with an earlier published method (Fekete *et al*., 2006). Briefly, 1 ml high-purity water was added onto 1 g of freeze-drying washed cell pellets (phase I and II with control) and sonicated for 15 min. After centrifugation with for 5 min the supernatant was directly injected into the CE (Beckmann P/ACE system 5500, Beckman Coulter, Germany) by pressure (0.5 psi for 10 s). The sample was then separated in fused silica capillary (Polymicro Technologies, Phoenix, AZ, USA) with dimension of 75 μm I.D and effective length of 40 cm, termostated at 30°C under voltage of 20 kV. The measured electropherograms at 214 nm were then transformed into mobility scale for more reliable quantification (Schmitt-Kopplin *et al*., 2001a,b). The concentrations were determined from peak area with calibration and the relative standard deviations were determined from three independent samples.

### Analysis of amino acids

The analysis of the glutamatic acid was performed by UPLC Acuity System (Waters, Milford, MA, USA) equipped with a 2996 PDA detector which was applied for analysis. The samples were the same as used for amine analyses; however, it had to be diluted (1:2). For better sensitivity and selectivity, the amino acids were derivatized with 6-aminoquinolyl-*N*-hydroxysuccinimidyl carbamate (Waters, Milford, MA, USA). The separation column was BEH C_18_ filled with packing material with 1.7 μm particle sizes (Waters, Milford, MA, USA) with dimensions of 2.1 × 100 mm, which was the type of the column. The column was thermostated at 35°C, the sample system at 27°C. Ten microlitres of sample was injected via partial-loop injection with needle overfill. The system was run with a linear solvent strength gradient starting with 0.2% acetic acid solution to 15% acetonitrile in 5 min. The flow rate was set to be 0.6 ml min^−1^ which resulted in a system pressure of 760 bars. Detection was performed at 260 nm at a scan rate of 20 Hz. The peak areas were calculated using Waters Empower software and the concentrations were determined by calibration.

### Ultra-high resolution mass spectrometry

High-resolution mass spectra for molecular formula assignment were acquired at GSF on a Bruker (Bremen, Germany) APEX Qe Fourier transform ion cyclotron resonance mass spectrometer equipped with a 12 Tesla superconducting magnet and an Apollo II ESI source. The samples were diluted in methanol and measured in negative electrospray mode. Samples were introduced into the microelectrospray source at a flow rate of 120 μl h^−1^ with a nebulizer gas pressure of 20 psi and a drying gas pressure of 15 psi (200°C). Spectra were externally calibrated on clusters of arginine (10 mg l^−1^ in methanol); calibration errors in the relevant mass range were always below 0.1 p.p.m. The spectra were acquired with a time domain of one megaword with a mass range of 100–600 m/z. The spectra were zero filled to a processing size of two megawords. The ion accumulation time in the ion source was set to 0.2 s for 1024 scans accumulated per samples.

## Supplementary material

The following supplementary material is available for this article online:

Fig. S1. Experimental design and current strategy used to identify genes modulated by Cd in phase I and phase II cells of Pseudomonas brassicacearum.

Table S1. Primers sequences, temperature and cycles number used for semi-quantitative RT-PCR.

This material is available as part of the online article from http://www.blackwell-synergy.com
