# Supplementary material for: Exploration of intraclonal adaptation mechanisms of Pseudomonas brassicacearum facing cadmium toxicity
Source: Environ Microbiol. 2007 Nov;9(11):2820–35. doi: 10.1111/j.1462-2920.2007.01394.x (PMC2121137; doi:10.1111/j.1462-2920.2007.01394.x)
Supplement: Fig. S1. — Experimental design and current strategy used to identify genes modulated by Cd in phase I and phase II cells of Pseudomonas brassicacearum. [file emi0009-2820-SD1.doc]

**Phase II** cells untreated

**Phase II** cells treated (CdCl2 25 μM)

Cy3

Hybridization 1 hyb1phaseII (.gpr)

**Phase II** cells untreated

**Phase II** cells treated (CdCl2 25 μM)

**Phase II** cells untreated

**Phase II** cells treated (CdCl2 25 μM)

**Phase II** cells untreated

**Phase II** cells treated (CdCl2 25 μM)

Cy5

Cy3

Cy5

Cy5

Cy3

Cy5

Cy3

Hybridization 2 hyb2phaseII (.gpr)

Hybridization 3 hyb3phaseII (.gpr)

Hybridization 4 hyb4phaseII (.gpr)

Replicate I

dye

swap

Replicate II

dye

swap

**Total RNA extraction**

**&**

**DNAse treatment**

**Labeling**

**Hybridization**
