# Supplementary material for: Exploration of intraclonal adaptation mechanisms of Pseudomonas brassicacearum facing cadmium toxicity
Source: Environ Microbiol. 2007 Nov;9(11):2820–35. doi: 10.1111/j.1462-2920.2007.01394.x (PMC2121137; doi:10.1111/j.1462-2920.2007.01394.x)
Supplement: Table S1. — Primers sequences, temperature and cycles number used for semi-quantitative RT-PCR. [file emi0009-2820-SD2.doc]

**Table S1 :** Primers sequences, temperature and cycles number used for semi quantitative RT-PCR.

| **Gene (accession number)** | **Primers** |
| --- | --- |
| *cadA* | FOR 5' TAG TGA TCC CAA GCA GCA CC 3' |
|  | REV 5' GAC CCA GCG CTT CGT CGA CC 3' |
| *lvs* | FOR 5' AGC CAT TGG TCA GTG CGG 3' |
|  | REV 5' CCG ACC AAC CAT CGA CGG AGG 3' |
| *alg8* | FOR 5' GTG GTG CTG TTC GAC CAG CG 3' |
|  | REV 5' AGG ACT GTT GAT CGA GGC 3' |
| *phlA* | FOR 5' TAT ACT GCA GGT GTA CTT CCT CCA GAT TCC 3' |
|  | REV 5' GAG AGT GCA GCG AAA ACA AAC CTA TTG TTC 3' |
| *phlB* | FOR 5' GCG GGG TGT AGC GGG CGC 3' |
|  | REV 5' CGG CAA GTT CGT CAG CGG 3' |
| *phlC* | FOR 5' GTG CAA AAT CCC ATC GCA ACC 3' |
|  | REV 5' GTA TGT GGT CCA GGC CAG GC 3' |
| *phlD* | FOR 5' TGA CCG CCT TGT CCA GGG TG 3' |
|  | REV 5' TGC GAG CCG ACG ATC AGG CG 3' |
| *potF1* | FOR 5' TCC GGT TAT GAC ATC GTG GTG 3' |
|  | REV 5' AGT TGG TCG GCG CGT CGG CA 3' |
| *potF2* | FOR 5' GGC ATC GGC TAC AAC GTC G 3' |
|  | REV 5' CAG GTA ATT GAG CAT CGC C 3' |
| *16S (control gene)* | FOR 5' AGA GTT TGA TCC TGG CTC AG 3' |
|  | REV 5' TCT ACG CAT TTC ACC GCT AC 3' |
